# The effects of pre- and postnatal depression in fathers: a natural experiment comparing the effects of exposure to depression on offspring

**DOI:** 10.1111/j.1469-7610.2008.02000.x

**Published:** 2008-10

**Authors:** Paul G Ramchandani, Thomas G O'Connor, Jonathan Evans, Jon Heron, Lynne Murray, Alan Stein

**Affiliations:** 1 Section of Child and Adolescent Psychiatry, University of OxfordUK; 2 Department of Psychiatry, University of RochesterNY, USA; 3 Academic Unit of Psychiatry, University of BristolUK; 4 Department of Community Based Medicine, University of BristolUK; 5 Winnicott Research Unit, Department of Psychology, University of ReadingUK

**Keywords:** Depression, child behavioural problems, perinatal, fathers, ALSPAC

## Abstract

**Background:**

Depression in fathers in the postnatal period is associated with an increased risk of behavioural problems in their offspring, particularly for boys. The aim of this study was to examine for differential effects of depression in fathers on children's subsequent psychological functioning via a natural experiment comparing prenatal and postnatal exposure.

**Methods:**

In a longitudinal population cohort study (the Avon Longitudinal Study of Parents and Children (ALSPAC)) we examined the associations between depression in fathers measured in the prenatal and postnatal period (measured using the Edinburgh Postnatal Depression Scale), and later behavioural/emotional and psychiatric problems in their children, assessed at ages 3½ and 7 years.

**Results:**

Children whose fathers were depressed in both the prenatal and postnatal periods had the highest risks of subsequent psychopathology, measured by total problems at age 3½ years (Odds Ratio 3.55; 95% confidence interval 2.07, 6.08) and psychiatric diagnosis at age 7 years (OR 2.54; 1.19, 5.41). Few differences emerged when prenatal and postnatal depression exposure were directly compared, but when compared to fathers who were not depressed, boys whose fathers had postnatal depression only had higher rates of conduct problems aged 3½ years (OR 2.14; 1.22, 3.72) whereas sons of the prenatal group did not (OR 1.41; .75, 2.65). These associations changed little when controlling for maternal depression and other potential confounding factors.

**Conclusions:**

The findings of this study suggest that the increased risk of later conduct problems, seen particularly in the sons of depressed fathers, maybe partly mediated through environmental means. In addition, children whose fathers are more chronically depressed appear to be at a higher risk of emotional and behavioural problems. Efforts to identify the precise mechanisms by which transmission of risk may occur should be encouraged to enable the development of focused interventions to mitigate risks for young children.

Children of parents with psychiatric disorders are at increased risk of disorder themselves, as well as other developmental difficulties (Goodman & Gotlib, 1999; Murray & Cooper, 2003). Depression is the most significant of these parental disorders in terms of worldwide impact (Patel, 2007). Its potential impact on children has also been studied extensively. There is particular interest in maternal depression in the postnatal period because of specific associations with children's behavioural, social and psychiatric problems (Goodman, Brogan, Lynch, & Fielding, 1993; Halligan, Murray, Martins, & Cooper, 2007) and, in the developing world, effects on physical health as well as socio-emotional development (Rahman, Iqbal, Bunn, Lovel, & Harrington, 2004; Tomlinson, Cooper, & Murray, 2005). The mechanisms underlying these associations have, by and large, not been clearly disentangled because of the confounded nature of genetic and psychosocial risks (Goodman & Gotlib, 1999).

Depression also affects men during the postnatal period and in later child-rearing years (Goodman, 2004), but the effects of paternal psychiatric disorder on children's development are under-studied (Kane & Garber, 2004), particularly during the early years of infant and early child development. In part this may be due to an underestimation of the impact of paternal influence on early child development (Cabrera, Tamis-LeMonda, Bradley, Hofferth, & Lamb, 2000; Lamb & Lewis, 2004), but it may also be because of the challenges of including men in child development research (Cassano, Adrian, Veits, & Zeman, 2006). Nevertheless, studies do demonstrate that psychiatric disorders affecting men in the perinatal period may predict disorder in their offspring. Specifically, depression in fathers in the postnatal period is associated with increased rates of subsequent behavioural problems in their children (Carro, Grant, Gotlib, & Compas, 1993; Ramchandani, Stein, Evans, & O'Connor, 2005; Ramchandani et al., 2008); associations appear stronger for boys, but this has not been confirmed. As in the case of maternal depression, there remains some uncertainty as to the mechanisms involved. In particular, the experience of exposure to an adverse psychosocial environment created by a depressed parent is typically confounded by exposure to genetic risk – a point repeatedly made by behavioural geneticists (Plomin, 1995). The aim of the current paper is to use a novel natural experiment that capitalises on a pre- postnatal design using fathers, to test the hypothesis that exposure to psychosocial factors can account for the link between paternal depression and children's behavioural problems.

## Rationale for a natural experiment comparing prenatal and postnatal exposure

The degree to which psychosocial risks operate directly through psychosocial mechanisms is a central conceptual and methodological question in developmental science. Resolving this matter has proved especially vexing in the context of research on parenting and family process. That is because links between psychosocial risks, such as parenting and family process, may not be mediated via psychosocial channels. In the current study of paternal depression we compare the effects of prenatal-only versus postnatal-only depression in fathers, and consider what additional effect exposure to postnatal depression may have on children's behavioural adjustment. In other words, we investigate if *direct* exposure is necessary to see the adverse effects of paternal depression on children.

There have been few previous studies utilising the prenatal-versus-postnatal design, but some comparable designs have been used. For example, studies (e.g., Collishaw, Dunn, O'Connor, & Golding, 2007) demonstrate that risks occurring (to the parent) prior to the child's birth predict child outcomes; the implication is that the effects of the particular risk (e.g., divorce, single-parenthood, leaving home early) are transmitted to the child not from psychological exposure (i.e., the child did not experience the risk themselves) but via other mechanisms. Murray provided one of the only pre- versus postnatal assessments of maternal depression, finding that children whose mothers were depressed only prenatally had comparable behavioural outcomes to children of non-depressed mothers, and both groups fared better than children of postnatally depressed mothers (Murray, 1992). The implication was that exposure to depression was *necessary* to observe adverse outcomes. We extend that work by examining fathers and by addressing the question in a large community cohort, ALSPAC, that includes depressed and nondepressed men in the prenatal and postnatal period. We reasoned that the prenatal versus postnatal design might be especially valuable for understanding the nature of risk mechanisms where it is difficult to disentangle the confluence of psychosocial, genetic and other sources of risk, as in the case of paternal depression.

In this natural experiment we distinguish between prenatal and postnatal paternal psychopathology and compare direct (postnatal) exposure to the risk – paternal depression – with indirect or latent (prenatal) exposure (in this case latent risk is assumed to include genetic risk and also those life course patterns that are known to distinguish depressed individuals (Rodgers, 1990)). The aim of this design is to provide additional leverage for testing the hypothesis that psychosocial factors, including but not limited to the parenting context, account for the link between paternal depression and children's outcomes.

Specifically, where both prenatal and postnatal data are available, four groups of interest can be formed: 1) children whose fathers were not depressed either prenatally or postnatally; 2) children whose fathers were depressed prenatally only; 3) children whose fathers were depressed postnatally only; and 4) children whose fathers were depressed both prenatally and postnatally. Separation into these four groups offers the opportunity to compare direct exposure with latent risk. The logic and key comparisons of the design are as follows:

- If paternal depression carries no risk for the child (via any risk mechanism), then there should be no difference between the non-depressed and any of the three depressed groups. This is the initial null hypothesis, which is an important baseline starting point.
- The second comparison is that between the non-depressed group and the prenatal-only and postnatal-only groups. If direct psychological exposure is necessary in order to see the effects of paternal depression, then the children in the postnatal-only group would show worse outcomes than children in the non-depressed and prenatal-only groups.
- The third comparison of interest is that between the prenatal only group and both the postnatal-only and the prenatal plus postnatal depressed groups, which provides an additional test of the exposure (i.e., both and postnatal-only > prenatal only) effect as well as of severity (non-depressed < prenatal only = postnatal only < prenatal plus postnatal), where the group depressed at both times might be expected to experience higher rates of chronic adversity and possibly genetic risk.

In order for these comparisons to reliably distinguish the effects of direct exposure from latent risk, several assumptions must be made. The most important is that the prenatal-only and postnatal-only groups do not differ in other areas of risk; that is, whether the ‘onset’ of depressive symptoms occurs prior to or following the child's birth is essentially random, with no discernible selection effects. Other issues, including the accurate measurement of depression and adequate length of follow-up, are important but perhaps more difficult to address directly; we consider these and other design considerations in the discussion.

In the present study we sought to explore these contrasts via a natural experiment in a large population cohort study, the Avon Longitudinal Study of Parents and Children (ALSPAC). Our focus on outcome is behavioural problems, the most widely cited consequence of parental psychopathology and for which the mediating mechanisms are still largely uncertain. Based on prior research (Ramchandani, Stein, Evans, & O'Connor, 2005), we focus on conduct problems and consider the outcomes for boys and girls separately. Finally, as a rigorous test of our hypotheses, we examine children's behavioural adjustment at both 3½ and 7 years.

## Methods

The Avon Longitudinal Study of Parents and Children (Golding, Pembrey, & Jones, 2001) is a large, population-based longitudinal study, centred on the city of Bristol, UK. The initial ALSPAC sample consisted of 14,541 pregnant women. Questionnaires were sent to mothers and fathers at regular points during and after pregnancy; of the 13,586 responding to the first questionnaire, 13,228 had partners. The mean age of the fathers was 28.8 years (standard deviation 9.75) and 96% were white in ethnic origin. One-fifth (18.2%) were educated to degree level. Most of the households (55.5%) had at least one additional child in them.

All participants provided informed consent, and ethical approval was obtained from the ALSPAC Law and Ethics Committee and Local Research Ethics Committees.

### Measures

Fathers were assessed in week 18 of their partners’ pregnancy and again at 8 weeks after the birth of their infant. The Edinburgh Postnatal Depression Scale (EPDS) was used to assess symptoms of depression. The EPDS is a well-validated, widely used, self-report questionnaire that consists of ten items (Cox, Holden, & Sagovsky, 1987). Scores of more than 12 identify a diagnosis of major depressive disorder in women with a high specificity (95.7%) and sensitivity (81.1%) (Murray & Carothers, 1990). The EPDS has also been used in a number of studies with men, using the same cut-off score of above 12. Estimates of sensitivity range from 71% to 86% and specificities range from 75% to 94% (Deater-Deckard, Pickering, Dunn, & Golding, 1998; Matthey, Barnett, Kavanagh, & Howie, 2001). We used scores of more than 12 with both mothers and fathers, to identify a depressed group.

At age 3½ years children's emotional and behavioural problems were measured using maternal reports on the Rutter Revised Preschool Scales (Elander & Rutter, 1996). Each item on this measure describes a characteristic or behaviour (e.g., ‘is worried, worries about many things’, ‘fights with other children’), with three possible responses – Yes certainly, Yes sometimes, No. Individual items combine to form three problem scales (emotional problems, conduct problems and hyperactivity), as well as a prosocial behaviours scale. All problem behaviours combine to give a total problems scale. In this study we used a cut-off of the top 10% of scores on each scale to define high-scorers. This cut-off has been used in previous research with this tool (Plomin, Price, Eley, Dale, & Stevenson, 2002). We also conducted some analyses using continuous scores from the Rutter questionnaires, and these are reported in the text. However generally, the cut-off scale scores (dichotomised variables) are presented here for consistency with other reports and because the higher levels of disturbance are of greater clinical significance.

The Development and Well-Being Assessment (DAWBA) questionnaire was completed by parents (usually mothers) and teachers about the children when they were 7 years old (Goodman, Ford, Richards, Gatward, & Meltzer, 2000). The questionnaire enquires about psychiatric symptoms and their resultant impact, generating DSM-IV psychiatric diagnoses. Parents and teachers are asked to provide written details of the nature of each of the symptoms, and how they impact on the child's functioning. The questionnaire responses are entered into a computer program which integrates the information from the two sources and provides likely diagnoses where appropriate. These are then assessed by experienced clinical raters. The DAWBA has been validated, and used in a National Survey of over 10,000 children and adolescents in the UK (Ford, Goodman, & Meltzer, 2003).

Questions were also asked to obtain the following information: 1) The age of the father at the time of the child's birth. 2) The number of other children in the family at the time of the child's birth. 3) Paternal educational level. 4) Paternal ethnicity. 5) Social class. 6) Marital status. 7) Past history of severe depression – self-reported (yes/no response).

### Statistical analysis

The analysis was undertaken in stages:

1. The sample was separated into four groups by paternal depression status: no depression at any time point, prenatal depression only (prenatal), postnatal depression only (postnatal), and depression at both times (the both group). The groups were then compared on a range of socio-demographic variables which may have acted as potential confounding factors.
2. We then conducted a series of planned contrasts to test any differential effects on children of exposure to paternal depression in the pre- and postnatal periods. The outcome in this first series of contrasts was high levels of emotional and behavioural symptoms in children at age 3½ years measured using the Rutter Revised Preschool Scales. We contrasted risk in the three depression groups (prenatal, postnatal, and both) using the not-depressed group as the reference (baseline) group, and then undertook planned analyses comparing the depressed groups with each other, to examine more specifically the contrasts outlined in the design.
  These same analyses were then repeated controlling for any potential confounding effect of maternal depression in the pre- and postnatal periods, and also for three other covariates found to differ between the depressed and non-depressed participants (paternal education level, marital status and other children in the family).
3. These analyses for Rutter Scale scores were then repeated for boys and girls separately. This was decided a priori.
4. These same analyses were then repeated excluding the small proportion of families where the biological father was no longer living with the family (12.9% of the sample), in order to exclude any stepfather confounds.
5. We repeated the original analyses using psychiatric status in the children at age 7 years as the outcome measure, to assess the persistence and developmental importance of any association.
6. Finally we undertook the analyses for outcomes at both 3 and 7 years using continuous depression scores at prenatal and postnatal times as predictors. The patterns of associations seen were very similar to those seen using logistic regression, and so those results are presented here for ease of interpretation. Results from the continuous analyses are available from the authors.

## Results

The results are presented in the same order as described in the methods section.

### Step 1

Questionnaires were completed by 7601 men at the two time points under study (18 weeks prenatal, and 8 weeks postnatal). Of these, 175 (2.3%) were depressed only at the prenatal time point (the prenatal group), 166 (2.2%) were depressed only at the postnatal time point (the postnatal group), and 89 (1.2%) were depressed at both times (the both group); 7171 (94.3%) were not depressed at either time. The non-depressed group were different from the three depressed groups in a number of ways: they had fewer children, were less likely to have a past history of depression and were more likely to be married than all three depressed groups. In addition the non-depressed group had more participants of white ethnicity than in the prenatal depressed group, and higher levels of educational attainment than the prenatal and the postnatal depressed groups. The only difference seen between the three depression groups was that the group depressed at both times had higher rates of a past history of depression than either the prenatal only or postnatal only groups. It is worth noting that there were no differences seen between the prenatal only and the postnatal only groups on a wide range of socio-demographic factors; in fact they appear very similar. These comparisons were rerun on the smaller group where child data was also available (*n* = 6449), and the same similarities and differences were found.

### Step 2

Children of fathers in all three depression groups had higher rates of total problems on the Rutter scales than children whose fathers had not been depressed (prenatal only 16.2% vs. 8.3%; unadjusted Odds Ratio 2.22 (95% Confidence Interval 1.34, 3.39); postnatal only 13.7% vs. 8.3%; unadj. OR 1.75 (1.07, 2.86); depressed at both times 24.3% vs. 8.3%; unadj. OR 3.55 (2.07, 6.08)) (see Table 2). There were no differences when the prenatal only and postnatal only groups were directly compared.

**Table:** Demographic features of the prenatal depression and postnatal depression groups

Factor	Not depressed (*n* = 7171)	Prenatal only (*n* = 175)	Postnatal only (*n* = 166)	Both group (*n* = 89)
Father age (mean & SD)	28.9 (9.6)	28.3 (8.8)	28.3 (10.2)	29.7 (10.8)
Number of children (as above)	.76 (.92)^a,b,c^	1.01 (1.05)	.99 (1.09)	1.20 (1.14)
Ed. level (% with degree)*	21.9^a,b^	17.0	17.2	15.9
Ethnicity (% white)	98.0^a^	95.3	98.8	96.5
Social class (% I&II)	49.6	44.7	46.2	40.3
Child gender (% girls)	48.9	46.3	48.8	49.4
Marital status (% married)	82.7^a,b,c^	64.5	73.8	72.4
Past history of depression (%)	4.6^a,b,c^	23.4^c^	22.0^c^	45.3

a = different from prenatal only group; b = different from postnatal only group; c = different from the ‘both’ group (all *p* < .05).

* Education level was analysed in 5 categories of attainment, although only the percentage gaining a degree is presented here for ease of reading.

**Table 2:** Paternal depression and child emotional and behavioural problems at age 3½ years (last 6 columns present inter-group contrasts (all unadjusted Odds Ratios (95% Confidence Intervals)))

Sub-scale of Rutter Q.	No depress. (*n* = 6100) *n* (%)	Pre-N (*n* = 136) *n* (%)	Post-N (*n* = 139) *n* (%)	Pre + Post-N (Both) (*n* = 74) *n* (%)	No. depress. vs. pre-N	No. depress. vs. Post-N	No. depress. vs. both	Pre-N vs. Post-N	Pre-N vs. Both	Post-N vs. Both
Emotional	787 (12.9%)	26 (19.1%)	24 (17.3%)	16 (21.6%)	**1.60 (1.04, 2.47)**	1.41 (.90, 2.21)	**1.87 (1.07, 3.27)**	.88 (.48, 1.63)	1.17 (.58, 2.35)	1.32 (.65, 2.68)
Conduct	640 (10.5)	19 (14.0)	20 (14.4)	13 (17.6)	1.39 (.85, 2.27)	1.44 (.89, 2.33)	**1.83 (1.00, 3.34)**	1.04 (.53, 2.04)	1.31 (.61, 2.84)	1.27 (.59, 2.72)
Hyperactivity	433 (7.1)	10 (7.4)	15 (10.8)	11 (14.9)	1.04 (.54, 2.00)	1.59 (.92, 2.74)	**2.29 (1.20, 4.38)**	1.52 (.66, 3.52)	2.20 (.89, 5.46)	1.44 (.63, 3.33)
Prosocial	549 (9.0)	10 (7.4)	13 (9.4)	11 (14.9)	.81 (.42, 1.54)	1.05 (.59, 1.87)	1.73 (.98, 3.07)	.91 (.45, 1.86)	1.67 (.76, 3.54))	1.83 (.85, 3.90)
Total problems	488 (8.3)	22 (16.2)	19 (13.7)	18 (24.3)	**2.13 (1.34, 3.39)**	**1.75 (1.07, 2.86)**	**3.55 (2.07, 6.08)**	.82 (.42, 1.60)	1.67 (.83, 3.36)	2.03 (.99, 4.16)

*p* < .05.

The group depressed at both times were more likely to have high scores than the non-depressed group on the emotional, conduct and hyperactivity symptoms scales. The prenatal-only group were more likely to have a high score than the non-depressed group on the emotional symptoms scale (19.1% vs. 12.9%; unadj. OR 1.60 (1.04, 2.47)). There were no statistically significant differences between the non-depressed group and either the prenatal or the postnatal groups on the conduct problems scale using the 10% cut-off, but differences were found when we used the continuous scale. With these scores, children in the postnatal-only depression-only group scored higher than the no-depression group, whereas the prenatal-only depression group did not (mean scores (S.D.) for the no-depression group was 3.54 (2.30), prenatal depression only 3.66 (2.47), and postnatal only 4.04 (2.44); *p* = .012)).

When we conducted the same contrasts controlling for the effects of maternal depression, paternal education level, marital status, and presence of other children, the differences seen previously were somewhat attenuated. For the whole sample, only the association seen between prenatal depression and total problems (OR 1.75 (1.06, 2.92)) and those depressed at both times and total problems (OR 2.39 (1.29, 4.47)) remained statistically significant; the association with postnatal depression did not (OR 1.45 (.86, 2.45)).

### Step 3

**Figure 1:**
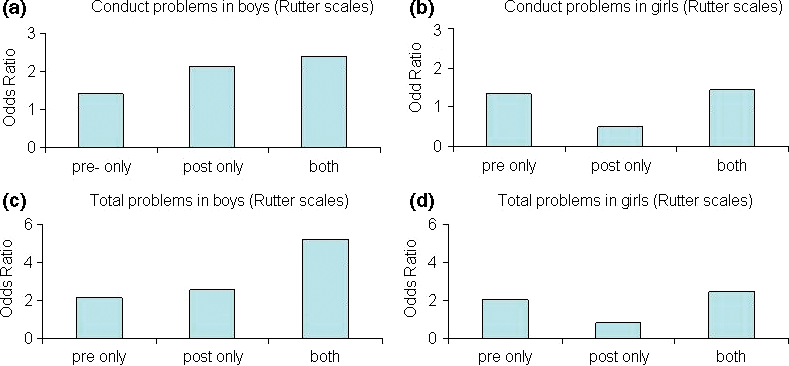
Comparison of conduct and total problems at age 3 years in the three depressed groups compared to the non-depressed group.

When we examined scores for boys and girls separately, similar patterns of difference emerged for the effects of paternal postnatal depression in boys, with increased rates of total problems seen in all three depressed groups, compared to the non-depressed group. The postnatal-only group and the group depressed at both times had higher rates of conduct problems. In contrast, prenatal depression was associated with higher scores on the emotional problems scale (see Table 3 and Figure 1), suggesting that the exposure hypothesis may be relevant for conduct problems in boys, but not for emotional or total problems.

**Table 3:** Paternal depression and child emotional and behavioural problems at age 3½ years (in boys and girls separately)

Sub-scale of Rutter Q.	No Depress. (*n* = 3118) (Boys) *n* (%)	Pre-N (*n* = 73) *n* (%)	Post-N (*n* = 74) *n* (%)	Pre + Post-N (Both) (*n* = 32) *n* (%)	Congruence with exposure hypothesis (Boys)	No Depress. (*n* = 2982) (Girls) *n* (%)	Pre-N (*n* = 63) *n* (%)	Post-N (*n* = 65) *n* (%)	Pre + Post-N (Both) (*n* = 42) *n* (%)	Congruence with exposure hypothesis (Girls)
Emotion.	387 (12.4%)	16 (21.9%)	13 (17.6%)	8 (25.0%)	Not congruent	396 (13.3%)	10 (15.9%)	11 (16.9%)	8 (19.0%)	Not congruent
Conduct	380 (12.2)	12 (16.4)	17 (23.0)	8 (25.0)	congruent	256 (8.6)	7 (11.1)	3 (4.6)	5 (11.9)	Not congruent
Hyper.	249 (8.0)	6 (8.2)	9 (12.2)	6 (18.8)	congruent	182 (6.1)	4 (6.3)	6 (9.2)	5 (11.9)	congruent
Prosocial	327 (10.5)	10 (13.7)	8 (10.8)	4 (12.5)	Not congruent	453 (15.2)	8 (12.7)	9 (13.8)	11 (26.2)	Not congruent
Total problems	283 (9.1)	13 (17.8)	15 (20.3)	11 (34.4)	congruent	223 (7.5)	9 (14.3)	4 (6.2)	7 (16.7)	Not congruent

For girls, fewer differences emerged, although those exposed to prenatal paternal depression and those whose fathers had depression at both times had higher rates of high total problems scores than those not exposed to paternal depression. A significant interaction was found between postnatal exposure to paternal depression and child gender for the outcome of conduct problems (*p* = .030). No other interactions by gender were found.

When the analyses were repeated controlling for confounding factors, we found that for boys postnatal depression (OR 2.44 (1.31, 4.56)) and depression at both times (OR 4.44 (2.03, 9.73)) remained associated with high total scores, whereas prenatal depression was not (OR 1.67 (.85, 3.27)). A similar pattern emerged for boys’ conduct problems, with the odds for postnatal depression (OR 2.30 (1.28, 4.11)) exceeding that for prenatal depression (OR .98 (.49, 1.97)).

### Step 4

When we repeated these analyses only in those families where the biological father was still living with the family at this time, the findings were largely unchanged. There were some small differences in the effects seen (see supplementary table). All contrasts between the depressed groups remained non-significant.

### Step 5

When the analyses were extended to include psychiatric diagnoses at age 7 years, a similar pattern of differences was found. All three depressed groups had higher rates of psychiatric diagnosis (any diagnosis) than the non-depressed group. The rates of anxiety disorders in the prenatal-only group were higher than in the non-depressed group, and postnatal paternal depression was more strongly associated with oppositional-conduct disorders (see Table 4). However, when we controlled for the effects of confounding variables, these associations attenuated somewhat and were no longer statistically significant (prenatal vs. no depression and anxiety disorders OR 2.00 (.84, 4.76); postnatal vs. no depression and oppositional/conduct disorders OR 1.72 (.73, 4.00)).

**Table 4:** Paternal depression and psychiatric problems in all children at age 7 years (last 6 columns present inter-group contrasts (all unadjusted Odds Ratios (95% Confidence Intervals)))

Disorder	No Depress. (*n* = 5195)	Pre-N (*n* = 117)	Post-N (*n* = 113)	Both (*n* = 58)	No. depress. vs. pre-N	No. depress. vs. post-N	No. depress. vs. Both	Pre-N vs. Post-N	Pre-N vs. Both	Post-N vs. Both	Congruence with exposure hypothesis
Anxiety	130 (2.5%)	7 (6.0%)	5 (4.4%)	3 (5.2%)	**2.52 (1.15, 5.53)**	1.83 (.74, 4.57)	2.16 (.67, 6.99)	.73 (.22, 2.36)	.86 (.21, 3.45)	1.18 (.27, 5.10)	Not congruent
Oppositional / Conduct	145 (2.8)	5 (4.3)	7 (6.3)	4 (6.9)	1.55 (.62, 3.86)	**2.32 (1.06, 5.08)**	2.57 (.92, 7.19)	1.49 (.46, 4.85)	1.66 (.43, 6.41)	1.11 (.31, 3.97)	Congruent
ADHD	99 (1.9)	3 (2.6)	1 (.9)	3 (5.2)	1.36 (.43, 4.37)	.47 (.07, 3.38)	2.83 (.87, 9.17)	.34 (.04, 3.34)	2.07 (.41, 10.64)	6.06 (.62, 58.82)	Not congruent
Depression	15 (.3)	0 (0)	1 (.9)	0 (0)	n/a	2.72 (.36, 20.41)	n/a	n/a	n/a	n/a	Not congruent
Any diagnosis	306 (5.9)	14 (12.0)	12 (10.7)	8 (13.8)	**2.16 (1.22, 3.82)**	**1.91 (1.04, 3.51)**	**2.54 (1.19, 5.41)**	.83 (.39, 2.00)	1.18 (.46, 2.99)	1.33 (.51, 3.47)	Not congruent

*p* < .05.

**Figure 2:**
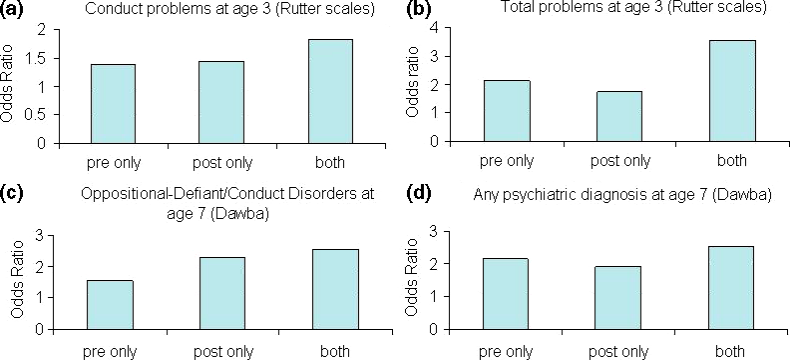
Comparison of conduct and total problems (age 3) and psychiatric diagnoses (age 7) in the three depressed groups compared to the non-depressed group.

The overall patterns of odds for conduct and total problems at ages 3 and 7 years can be more clearly seen in Figure 2. The pattern of odds for oppositional-defiant and conduct disorders appears congruent with an exposure hypothesis, whereas the pattern for overall problems and any diagnoses does not.

## Discussion

Our aim in this special issue on perinatal influences on children's health is to consider not only the substantive nature of perinatal paternal influences, but also to consider how a focus on the perinatal period may offer methodological and conceptual clarity for understanding influences on child development more broadly. We tested a psychosocial exposure natural experiment model by comparing the behavioural adjustment in children whose fathers were non-depressed, prenatally depressed, postnatally depressed, or depressed throughout the perinatal period. We found modest support for the psychosocial exposure hypothesis, particularly for conduct problems and in boys. We also found that children whose fathers were more chronically depressed had higher overall risks for adverse outcomes. Before turning to a discussion of these findings we first consider the limitations and strengths of the study, particularly in light of our proposed natural experiment.

This study has a number of strengths. It includes longitudinal data from a large unselected population sample, which has been followed up from before birth to the age of 7 years. We were able to use well-validated measures to assess parental and child psychopathology. Furthermore, paternal symptoms of depression and outcomes in children were rated by different people, as mothers rated the child's psychopathology in almost all cases, thereby substantially reducing the risk of rater bias. Finally, we were able to control for the potential confounding effects of maternal depression and other relevant socio-demographic variables, and to conduct sensitivity analyses to assess any effect of excluding families where the biological father was not present through the first three years of the child's life.

There were a number of limitations to the study. The numbers in each of the separate depression groups, while reasonable overall (*n*'s = 89, 166 and 175 in the depressed groups), are small in the context of the overall population (*n* = 7601). This reduced the power of the study to find differences between the groups – particularly between the depressed groups. Second, we were unable to use a diagnostic assessment of paternal depression, although the instrument used, the EPDS, does have good sensitivity and specificity for a diagnosis of depression. Third, the use of a cut-off score for depression means that there is a possibility of some overlap between the groups depressed in the prenatal and postnatal periods, as depression may be both cyclical and also demonstrate considerable continuity. A participant scoring just below the cut-off at one time point and just above at the other may not have had significantly different psychopathology at the two time points, but would be rated as not depressed at one point and depressed at the other. We attempted to address this in part by repeating the analyses using continuous depression scores. The similarity of the findings, independent of the method of analysis used, suggests that the use of cut-offs did not result in artefactual findings.

This study represents an early attempt to disentangle direct from latent effects of paternal depression on the development of their children. Negative findings (findings of no difference between the groups) are difficult to draw clear conclusions from, as some of the groups had relatively small numbers. The finding of similar odds ratios in the prenatal only and postnatal only groups for high total problem scores at age 3 years, and for any psychiatric diagnosis at age 7 years, may point to latent risks accounting for the increased rates of these difficulties seen where fathers experience depression, but without confirmatory studies, firm conclusions cannot be drawn. It is somewhat clearer that when oppositional defiant and conduct disorders alone are considered, the postnatal group and the group depressed at both times have the highest rates of disorder. This is also the case when boys are considered alone for both conduct and total problems. The evidence suggests that for behavioural problems and disorders, perhaps particularly in boys, active exposure to the effects of paternal depression may be significant, over and above the latent risks transmitted. While the design was useful in providing a test of the exposure hypothesis, further study is required to discern which specific psychological processes and exposures might account for the increased rate of disturbance.

The use of natural experiments incorporating prenatal and postnatal exposure allows a broader range of research questions to be addressed than studies using just one exposure period. These include the examination of the mechanisms of risks for other parental and family exposures such as other forms of parental ill-health, the effects of divorce or parental separation (for example due to imprisonment). There have been relatively few studies of this kind (Kim-Cohen, Moffitt, Taylor, Pawlby, & Caspi, 2005; Murray & Farrington, 2005; Murray, 1992), and they can be difficult to conduct, requiring longitudinal follow-up, and large numbers of participants, depending on the frequency of the exposure of interest. Nevertheless, they offer promise for disentangling some important questions of considerable interest for developmental psychopathology.

One other aspect of this study deserves further mention; the study of paternal psychiatric disorders is still relatively unusual, although there is a developing body of research in the field (e.g., Cummings, Keller, & Davies, 2005; Paulson, Dauber, & Leiferman, 2006). The study of fathers may give unique insights which are not apparent when studying only mothers (Cabrera, Tamis-LeMonda, Bradley, Hofferth, & Lamb, 2000). In this instance, it would be difficult to conduct the same study with mothers because of the strong evidence from both animal and human research that increased maternal stress during pregnancy can have a direct impact on foetal development. This means that prenatal exposure through mothers would not be able to be largely considered a non-environmental exposure in the way that prenatal paternal psychopathology might. However, it should of course be remembered that prenatal paternal disorders may also impact upon a developing foetus through an associated increase in maternal stress (O'Connor, Heron, Golding, & Glover, 2003), although we were able to control for maternal depression during pregnancy in this study.

In conclusion, the findings of this study suggest that the mechanisms underlying the association between depression in fathers and subsequent emotional/behavioural and psychiatric outcomes in their children may vary depending on the outcome being measured. The findings point to the possibility that the increased risk of later conduct problems in the sons of depressed fathers maybe partly mediated through environmental means. There is less clear evidence where risks for more general psychopathology are concerned. Children of fathers with more persistent depression have higher risks of subsequent emotional and behavioural problems. Efforts to identify the precise psychological mechanisms by which risk transmission occurs should be sustained and encouraged to enable the potential identification and development of interventions to mitigate risks for developing infants and young children.

## Abbreviations:

ALSPAC: the Avon Longitudinal Study of Parents and Children
DAWBA: Development and Well-Being Assessment

## Supporting information

**Supplementary table.** Only biological dad present. (Word document)
