# Supplementary material for: The effects of pre- and postnatal depression in fathers: a natural experiment comparing the effects of exposure to depression on offspring
Source: J Child Psychol Psychiatry. 2008 Oct;49(10):1069–78. doi: 10.1111/j.1469-7610.2008.02000.x (PMC2737608; doi:10.1111/j.1469-7610.2008.02000.x)
Supplement: Supplementary file 1 [file jcpp0049-1069-SD1.doc]

**Supplementary table** (Only biological dad present)

| Sub-scale of Rutter Q. | No. depress. vs. pre-N | No. depress. vs. post-N | No. depress. vs. both | Pre-N vs. Post-N | Pre-N vs. Both | Post-N vs. Both |
| --- | --- | --- | --- | --- | --- | --- |
| Emotion. | 1.46 (0.87, 6.03) | **1.70 (1.03, 2.83)** | **2.36 (1.22, 4.56)** | 1.16  (0.56, 2.39) | 1.61  (0.70, 3.71) | 1.39  (0.61, 3.16) |
| Conduct | 1.25 (0.68, 2.30) | 1.15 (0.61, 2.16) | **2.39 (1.18, 4.82)** | 0.92  (0.38, 2.19) | 1.91  (0.76, 4.80) | 2.09  (0.82, 5.31) |
| Hyper. | 1.38 (0.69, 2.76) | 1.57 (0.81, 3.04) | **3.18 (1.53,6.63)** | 1.14  (0.44, 2.93) | 2.31  (0.85, 6.26) | 2.03  (0.77, 5.39) |
| Prosocial | 1.05 (0.58, 1.89) | 1.36 (0.79, 2.33) | 1.39 (0.65, 2.98) | 1.29  (0.56, 2.85) | 1.32  (0.51, 3.45) | 1.03  (0.41, 2.60) |
| Total problems | **2.19 (1.22, 3.74)** | 1.67 (0.91, 3.08) | **3.56 (1.80, 7.03)** | 0.78  (0.35, 1.77) | 1.67  (0.70, 3.97) | 2.13  (0.86, 5.26) |
